# MicroRNA-24 Attenuates Neointimal Hyperplasia in the Diabetic Rat Carotid Artery Injury Model by Inhibiting Wnt4 Signaling Pathway

**DOI:** 10.3390/ijms17060765

**Published:** 2016-05-24

**Authors:** Jian Yang, Zhixing Fan, Jun Yang, Jiawang Ding, Chaojun Yang, Lihua Chen

**Affiliations:** 1 Department of Cardiology, the First College of Clinical Medical Sciences, China Three Gorges University, Yichang 443000, China; yangjunyichang@163.com (J.Y.); dingjiawang@ctgu.edu.cn (J.D.); 2 Institute of Cardiovascular Diseases, China Three Gorges University, Yichang 443000, China; fanzhixing@ctgu.edu.cn (Z.F.); yangchaojun@ctgu.edu.cn (C.Y.); 3 Department of Optometry and Ophthalmology, Yichang Central People’s Hospital, Yichang 443000, China; chenlihua@ctgu.edu.cn

**Keywords:** hyperglycemia, carotid artery balloon injury, vascular restenosis, vascular smooth muscle cells, neointimal hyperplasia, microRNA-24

## Abstract

The long-term stimulation of hyperglycemia greatly increases the incidence of vascular restenosis (RS) after angioplasty. Neointimal hyperplasia after vascular injury is the pathological cause of RS, but its mechanism has not been elucidated. MicroRNA-24 (miR-24) has low expression in the injured carotid arteries of diabetic rats. However, the role of miR-24 in the vascular system is unknown. In this study, we explore whether over-expression of miR-24 could attenuate neointimal formation in streptozotocin (STZ)-induced diabetic rats. Adenovirus (Ad-miR-24-GFP) was used to deliver the *miR-24* gene to injured carotid arteries in diabetic rats. The level of neointimal hyperplasia was examined by hematoxylin-eosin (HE) staining. Vascular smooth muscle cell (VSMC) proliferation in the neointima was evaluated by immunostaining for proliferating cell nuclear antigen (PCNA). The mRNA levels of miR-24, PCNA, wingless-type MMTV integration site family member 4 (Wnt4), disheveled-1 (Dvl-1), β-catenin and cell cycle-associated molecules (Cyclin D1, p21) were determined by Quantitative Real-Time PCR (qRT-PCR). PCNA, Wnt4, Dvl-1, β-catenin, Cyclin D1 and p21 protein levels were measured by Western blotting analysis. STZ administration decreased plasma insulin and increased fasting blood glucose in Sprague-Dawley (SD) rats. The expression of miR-24 was decreased in the carotid artery after a balloon injury in diabetic rats, and adenoviral transfection (Ad-miR-24-GFP) increased the expression of miR-24. Over-expression of miR-24 suppressed VSMC proliferation and neointimal hyperplasia in diabetic rats at 14 days. Furthermore, compared with Sham group, the mRNA and protein levels of PCNA, Wnt4, Dvl-1, β-catenin, and Cyclin D1 were strikingly up-regulated in the carotid arteries of diabetic rats after a balloon injury. Interestingly, up-regulation of miR-24 significantly reduced the mRNA and protein levels of these above molecules. In contrast, the change trend in p21 mRNA and protein levels was opposite after a balloon injury. However, over-expression of miR-24 after gene delivery increased the mRNA and protein levels of p21. We conclude that over-expression of miR-24 could attenuate VSMC proliferation and neointimal hyperplasia after vascular injuries in diabetic rats. This result is possibly related to the regulation of the expression of Cyclin D1 and p21 through the Wnt4/Dvl-1/β-catenin signaling pathway.

## 1. Introduction

Diabetes mellitus (DM) is a worldwide health problem in both developed and developing countries [1]. In 2013, the International Diabetes Federation (IDF) showed that adult DM had reached 300 million cases [2]. The improper control of diabetes will lead to a variety of serious complications, such as cardiovascular diseases, kidney failure, and blindness [3]. In addition, DM patients also have a higher incidence of vascular restenosis (RS) after angioplasty than non-diabetic patients [4].

As an independent risk factor for vascular RS, hyperglycemia could impair vascular endothelial barrier function and promote vascular RS after percutaneous coronary intervention (PCI) [4]. The pathological process of vascular RS is complex, including inflammation, focal thrombosis, extracellular matrix production, cellular proliferation and neointimal hyperplasia [5]. A large number of experimental and clinical studies have demonstrated that neointimal hyperplasia after vascular injury played a critical step in the process of vascular RS [6]. This process involves numerous molecular signaling cascades governing vascular smooth muscle cell (VSMC) migration, differentiation and proliferation [7]. Among these multiple factors, VSMC proliferation is a key event [8]. Briefly, when under healthy and unstimulated conditions, VSMCs are at a low proliferative rate, with a quiescent and contractile phenotype. However, VSMCs can migrate into the intimal layer from the medial layer after vascular injury, accompanied by a switch to a proliferative synthetic phenotype [8]. Previous experiments also have confirmed that some drugs could ease neointimal hyperplasia after PCI by inhibiting VSMC proliferation [9].

MicroRNAs (miRs) could regulate diverse cellular functions by subsequent mRNA degradation or translation inhibition [10]. Recent studies have identified that many miRs [11], such as miR-1, miR-21, miR-29, miR-31, miR-143/145, and miR-221/222, play important roles in neointimal hyperplasia by regulating the functions of VSMCs. Interestingly, Ji and his colleagues indicated that miR-24 sharply decreased in rat balloon injured carotid arteries [12]. In addition, our unpublished data has suggested that miR-24 is VSMC-enriched, but has low expression in endothelial cells (ECs). To sum up, the above research results demonstrated that miR-24 may be involved in the formation of neointimal hyperplasia and vascular RS by regulating the functional state of VSMCs instead of ECs.

Wnt proteins, first identified in drosophila, play an important role in embryogenesis and development. Recent studies have shown that the Wnt signaling pathway may also play a role in neointimal hyperplasia by regulating VSMC proliferation, migration and survival [13]. Previous studies have shown that the proteins Wnt2 and Wnt4 increased at the genetic level in the VSMC proliferation process, but only the altered expression of Wnt4 has a key effect [14]. Therefore, Wnt4 was the focus in the present research. As an important signaling molecule, β-catenin could enter into the nucleus and activate the downstream signaling molecules of the Wnt4 signaling pathway, including p21, Cyclin D1, and matrix metalloproteinase (MMP) [15]. It has been reported that Wnt4/β-catenin signaling induced VSMC proliferation and was associated with neointimal hyperplasia [16]. Disheveled (Dvls) are cytoplasmic scaffold proteins (including Dvl-1, Dvl-2, Dvl-3) that play an important role not only in β-catenin-dependent Wnt signaling pathway, but also in β-catenin-independent pathway [17]. Hua *et al.* have indicated that the Wnt4/β-catenin signaling pathway modulated vascular RS via Dvl-1 [16,18]. Therefore, we also took Dvl-1 into consideration in this study. In general, Wnt4-triggered signaling pathways were involved in VSMC proliferation and neointimal hyperplasia after vascular injury.

In fact, Wnt4 has been predicted as a potential target gene of miR-24 by the bioinformatics of TargetScan. In this study, we used adenovirus to deliver miR-24 into the balloon injured carotid artery to investigate its effect on neointimal hyperplasia in diabetic rats. The main aim of this study was to investigate the role of miR-24 in VSMC proliferation and neointimal formation in diabetic rats after a vascular injury. In this study, we (1) observe the changes in miR-24 in diabetic rat carotid arteries after a balloon injury; (2) determine if over-expression of miR-24 could attenuate VSMC proliferation and neointimal hyperplasia *in vivo*; (3) seek a better understanding of the underlying mechanism of miR-24 involved in these biological processes.

## 2. Results

### 2.1. Adenovirus Was Transfected into the Carotid Artery in Diabetic Rats Successfully

**Figure 1:**
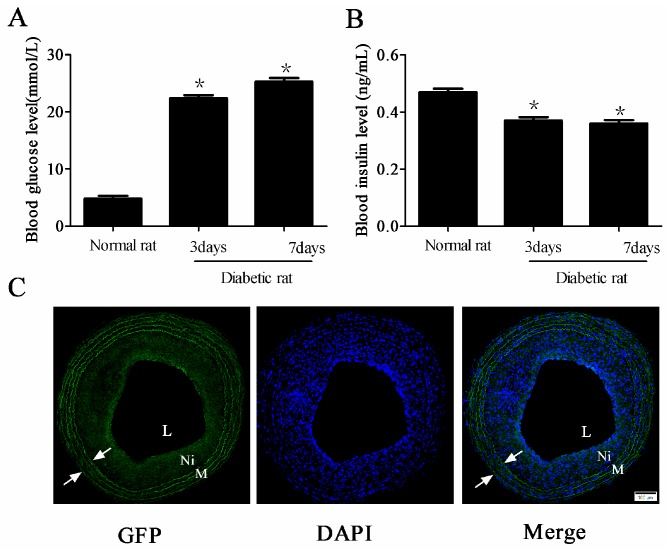
Adenovirus transfection into the carotid artery in diabetic rats successfully at two weeks after balloon injury. (**A**,**B**) The changes of fasting blood glucose (**A**) and plasma insulin (**B**) levels after injection of streptozotocin (STZ) (*n* = 10 in normal rat group, *n* = 50 in diabetic rat group, * *p* < 0.05 *vs.* normal rat group); and (**C**) Representative microphotographs of fluorescent microscopy after adenovirus transfection (100× magnification). Green fluorescent protein (GFP) and nuclei with 4′,6-diamidino-2-phenylindole (DAPI) were labelled with green and blue fluorescence, respectively. Arrows indicate the elastic lamina; L: Lumen; M: Media; Ni: Neointima. Scale bar represents 100 μm.

The diabetes model was established successfully. In brief, at the third day after injection of streptozotocin (STZ), the levels of fasting blood glucose increased, and compared with the normal rat, the blood glucose levels in diabetic rats continued to rise for seven days (* *p* < 0.05 *vs.* normal rat group, Figure 1A). However, the plasma insulin in diabetic rats decreased slightly (* *p* < 0.05 *vs.* normal rat group, Figure 1B). At two weeks post-transfection of adenovirus into the balloon-injured carotid artery in diabetic rats, fluorescent microscopy showed the emission green fluorescence, indicating that adenovirus transfection was successful (Figure 1C).

### 2.2. miR-24 Expression Was Decreased in Carotid Artery after Balloon Injury in Diabetic Rats

**Figure 2:**
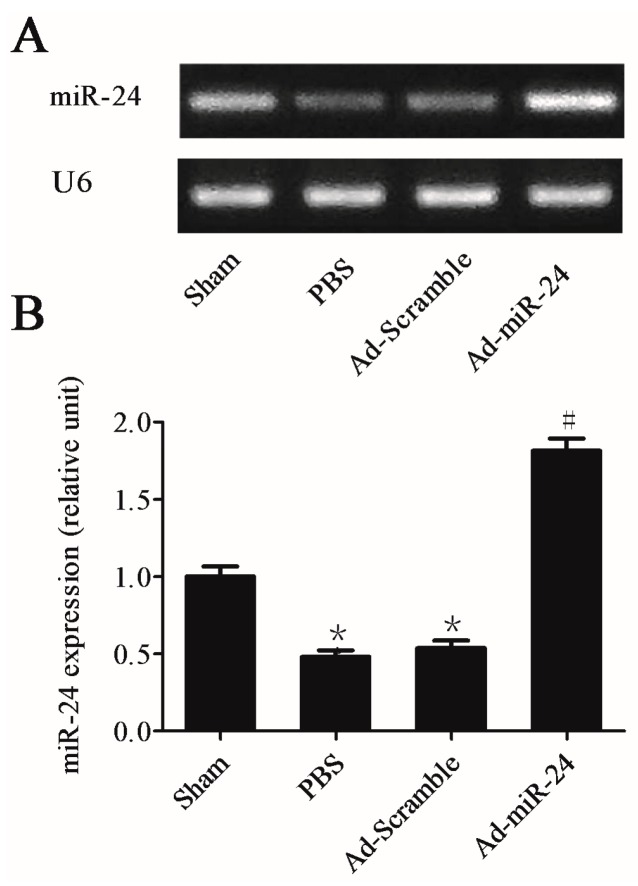
The expression level of microRNA-24 (miR-24) in carotid arteries. (**A**,**B**) Quantitative Real-Time PCR (qRT-PCR) gel (**A**) and quantitation (**B**) from the four groups for U6 (internal control of miRNA) and miR-24. The carotid artery balloon injury induced down-regulation expression of miR-24, while adenovirus (Ad-miR-24) transfection up-regulated its expression level (Compared to Sham group, * *p* < 0.05; Compared to phosphate buffer saline (PBS) group or Ad-Scramble group, ^#^ *p* < 0.05, *n* = 6). However, compared with the PBS group, Ad-Scramble could not increase the expression of miR-24.

miR-24 expression was examined both in uninjured and injured carotid arteries in diabetic rats. Two weeks after injury, compared to the uninjured control, the level of miR-24 from the balloon-injured carotid arteries were markedly reduced *vs*. phosphate buffer saline (PBS) group * *p* < 0.05. Interestingly, transfection with adenoviral over-expression of miR-24 into a balloon-injured carotid artery markedly increased miR-24 expression (adenovirus (Ad-miR-24) group *vs.* PBS group or Ad-Scramble group, ^#^ *p* < 0.05). However, compared with the PBS group, Ad-Scramble could not increase the level of miR-24 (Figure 2A,B, *n* = 6).

### 2.3. Over-Expression of miR-24 Suppressed Neointimal Hyperplasia in Diabetic Rats

**Figure 3:**
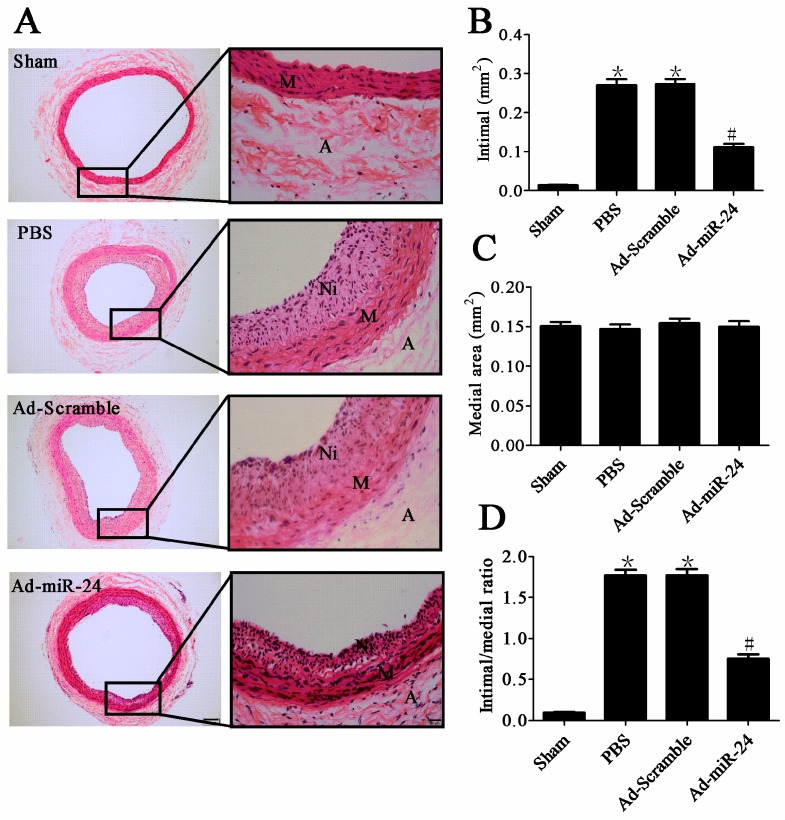
Effects of miR-24 on neointimal formation at two weeks after vascular injury and gene delivery: (**A**) Representative cross-sections of hematoxylin-eosin (HE) in different groups. Left (100× magnification, scale bar represents 100 μm), right (400× magnification, scale bar represents 20 μm); (**B**,**C**) Quantitative analysis of the neointimal formation (compared to Sham group, * *p* < 0.05; compared to PBS group or Ad-Scramble group, ^#^ *p* < 0.05, *n* = 6); (**D**) Ratio of intima to media (compared to Sham group, * *p* < 0.05; Compared to PBS group or Ad-Scramble group, ^#^ *p* < 0.05, *n* = 6). A: Adventitia; M: Media; Ni: Neointima.

Two weeks after the balloon injury and adenovirus transfection, the degree of neointimal hyperplasia was evaluated morphologically and quantitatively (Figure 3A). Ad-miR-24 treatment significantly reduced the neointimal area compared with Ad-Scramble and PBS (0.112 ± 0.007 *vs.* 0.275 ± 0.018 or 0.269 ± 0.024 mm^2^, ^#^ *p* both <0.05, Figure 3B, *n* = 6). The intima/media ratio was also markedly less in Ad-miR-24 transfected arteries (0.696 ± 0.056) than in Ad-Scramble transfected (1.702 ± 0.126, ^#^ *p* < 0.05, *vs.* Ad-miR-24 group) and PBS treated arteries (1.711 ± 0.131, ^#^ *p* < 0.05, *vs.* Ad-miR-24 group) (Figure 3D, *n* = 6). No significant differences in the medial area were observed between any of the groups (Figure 3C, *n* = 6).

### 2.4. Over-Expression of miR-24 Inhibited VSMC Proliferation in Diabetic Rats

**Figure 4:**
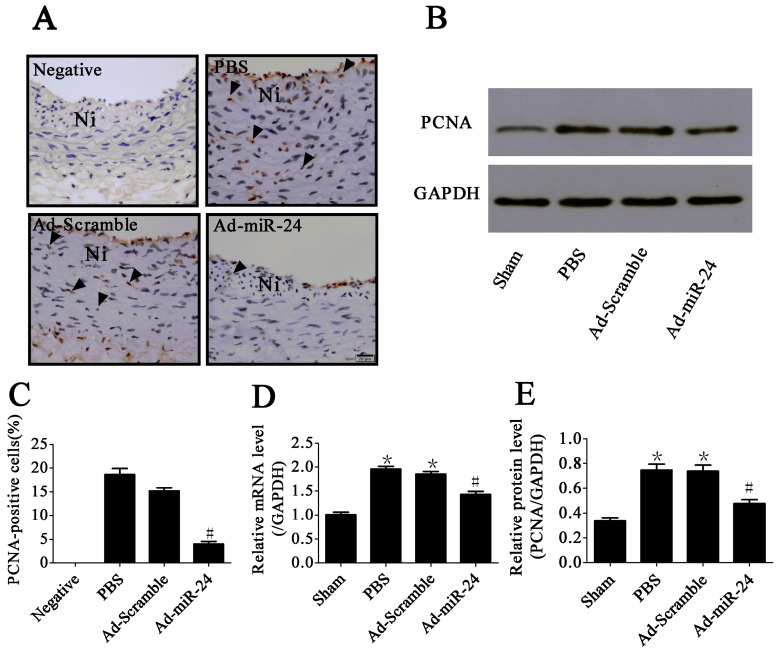
Anti-proliferative effect of miR-24: (**A**) Representative microphotographs of immunohistochemistry for proliferating cell nuclear antigen (PCNA) (400× magnification). Arrows indicate positive cells for PCNA. Ni: Neointima. Scale bar represents 20 μm; (**B**) Representative immunoblots of PCNA; (**C**) PCNA-positive rate in the neointima (compared to Ad-Scramble group and PBS group, ^#^ *p* < 0.05, *n* = 6); (**D**) The mRNA level of PCNA in carotid artery (compared to Sham group, * *p* < 0.05; compared to PBS group or Ad-Scramble group, ^#^ *p* < 0.05, *n* = 6); (**E**) Bar graphs of corresponding densitometric analyses of Western blots (compared to Sham group, * *p* < 0.05; compared to PBS group or Ad-Scramble group, ^#^ *p* < 0.05, *n* = 6).

Immunostaining for proliferating cell nuclear antigen (PCNA) was used to examine VSMC proliferation in the neointima. Compared with Ad-Scramble- and PBS-treated control groups, it could induce a significant reduction in the PCNA-positive cells in the neointima after treatment with Ad-miR-24 (4.5% ± 0.9% *vs.* 15.7% ± 3.9% and 18.4% ± 4.9%, respectively, ^#^ *p* both <0.05, Figure 4A,C, *n* = 6). Moreover, we also detected the mRNA and protein levels of PCNA using qRT-PCR and Western blotting and the result was consistent with the results of immunostaining (Figure 4B,D,E, *n* = 6).

### 2.5. Over-Expression of miR-24 Suppressed the Expression of Wnt4 Signaling Pathway

**Figure 5:**
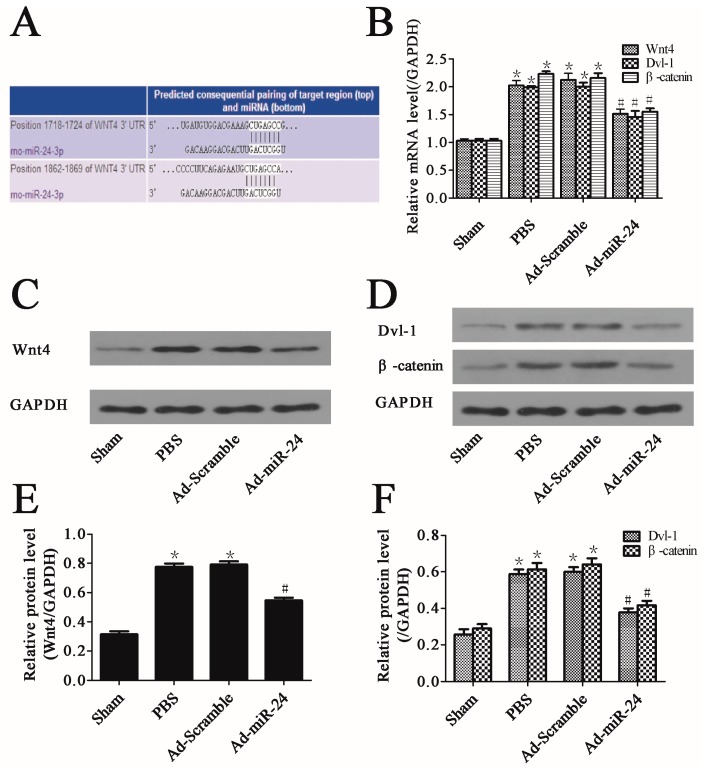
miR-24 over-expression suppressed the Wnt4 (protein)-triggered signaling pathway: (**A**) The mRNA of Wnt4 is a target of miR-24 predicted by using software TargetScan; (**B**) The mRNA levels of Wnt4, disheveled-1 (Dvl-1) and β-catenin in carotid artery (compared to Sham group, * *p* < 0.05; compared to PBS group or Ad-Scramble group, ^#^ *p* < 0.05, *n* = 6); (**C**) Representative immunoblot of Wnt4; (**D**) Representative immunoblots of Dvl-1and β-catenin; (**E**,**F**). Bar graphs of corresponding densitometric analyses of Western blots (compared to Sham group, * *p* < 0.05; compared to PBS group or Ad-Scramble group, ^#^ *p* < 0.05, *n* = 6).

Wnt4 has been predicted as a potential target gene of miR-24 with TargetScan software (Figure 5A) [19]. In this study, to determine whether miR-24 could modulate the activity of Dvl-1 and β-catenin through the regulation of Wnt4, we measured the mRNA and protein levels of Wnt4 signaling pathway-related molecules by qRT-PCR, Western blotting and densitometric analysis. Only in the Sham group did Wnt4, Dvl-1 and β-catenin have low expression levels. The balloon-injured carotid artery shared elevated levels of Wnt4 and downstream molecules both in gene and protein levels. The balloon injury plus adenoviral transduction of Ad-Scramble had no significant effect on the mRNA and protein levels of Wnt4, Dvl-1 and β-catenin. Interestingly, up-regulation of miR-24 could not only reduce gene expressions (Figure 5B, *n* = 6) but significantly reduce the levels of proteins by 36.07%, 34.67%, and 31.06%, respectively, in balloon-injured carotid arteries (Figure 5C–F, *n* = 6).

### 2.6. Over-Expression of miR-24 Altered the Expression of Cell Cycle-Associated Molecules

**Figure 6:**
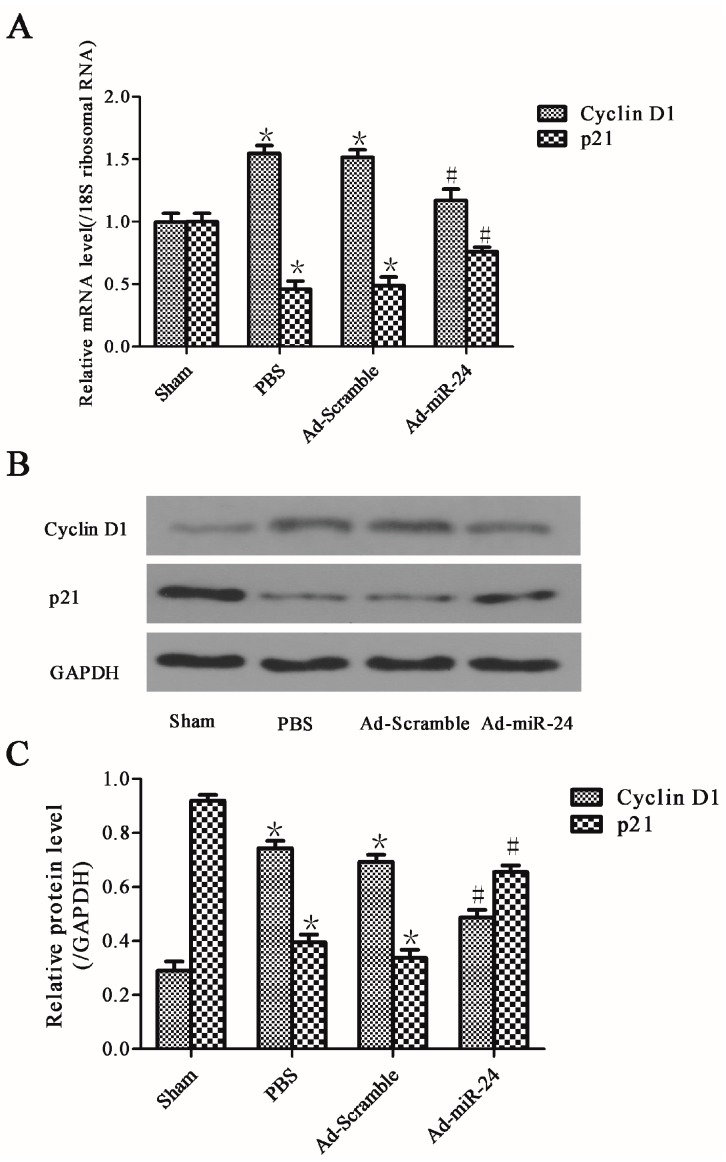
Effects of miR-24 over-expression on cell cycle-associated molecules: (**A**) The mRNA levels of Cyclin D1 and p21; (**B**) Representative immunoblots of cell cycle-associated molecules Cyclin D1 and p21; (**C**) Bar graphs of corresponding densitometric analyses of Western blots (compared to Sham group, * *p* < 0.05; compared to PBS group or Ad-Scramble group, ^#^ *p* < 0.05, *n* = 6).

To further analyze the exact mechanisms by which miR-24 inhibited VSMC proliferation and neointimal hyperplasia in the diabetic rats after vascular injury, the mRNA and protein levels of cell cycle-associated molecules were measured by Western blotting and qRT-PCR. Cyclin D1 and p21, as two key cell cycle-associated molecules, have been measured in this study. In the PBS group, both the gene and protein expressions of Cyclin D1 were increased, but p21 was repressed (PBS group *vs.* Sham group, * *p* both <0.05). Interestingly, over-expression of miR-24 significantly inhibited the gene and protein levels of Cyclin D1 but increased the levels of p21 (*vs*. Ad-Scramble group or PBS group, ^#^ *p* both <0.05). Additionally, a balloon injury followed by adenoviral transduction using Ad-Scramble had no significant effect on the levels of Cyclin D1 and p21 (Figure 6A–C, *n* = 6).

## 3. Discussion

Although the vascular RS rate has been reduced dramatically with the application of drug eluting stents (DES), it still plays a significant role in the long-term prognosis after angioplasty (such as PCI) [20]. Additionally, clinical evidence has indicated that the long-term stimulation of hyperglycemia greatly increases the incidence of vascular RS in diabetes [4,20]. It is necessary to carry out more intensive studies in the genetic mechanisms of vascular RS to ultimately resolve this problem [16]. Neointimal hyperplasia after vascular injury is the pathological cause of RS, but its mechanism has not been elucidated. In this study, we find that over-expression miR-24 by adenoviral transfection could reduce neointimal hyperplasia significantly in the injured carotid arteries of diabetic rats. Additionally, we demonstrated that over-expression of miR-24 could ease neointimal hyperplasia through its anti-proliferative effect on VSMCs. Our results also indicated that this anti-proliferative effect of miR-24 occurred via the suppression of the Wnt4 signaling pathway.

A number of miRs, such as, miR-1, miR-21, miR-29, miR-31, miR-143/145 and miR-221/222, were verified to be involved in neointimal hyperplasia by regulating the functions of VSMCs [11]. MiR-24 is a type of tumor-suppressing microRNAs [21]. Recently, Chan *et al.* also indicated that miR-24 could regulate the VSMC phenotype through the platelet derived growth factor (PDGF) and transforming growth factor-β (TGF-β) pathways [22]. However, the role of miR-24 in the neointimal formation of the diabetic rat after vascular injury remains unclear. In this present study, we found that miR-24 was significantly down-regulated in diabetic rat carotid arteries after a balloon injury at 14 days. We delivered miR-24 into rat carotid arteries successfully with the adenoviral vector-mediated transfection system. Over-expression of miR-24 reduced VSMC proliferation and neointimal hyperplasia in diabetic rats after vascular injury. Thus, we presume that miR-24 would be involved in the neointimal hyperplasia and it may have an anti-proliferative effect on VSMCs.

miR-24 is a VSMC-enriched microRNA and it has several target genes in VSMCs predicted by TargetScan. Among all the target genes, the *Wnt4* gene has attracted attention due to its characteristic of being closely related to cell proliferation [17]. In this study, our experiments confirmed that the expressions of miR-24 and Wnt4 were inversely correlated in diabetic rat carotid arteries. Wnt signaling has a recognized role in controlling the proliferation of cells in human cancers, and its role in VSMCs has also been considered recently [16,18]. Tsaousi *et al.* have revealed that the specific Wnt protein, Wnt4, is involved in the regulation of VSMC proliferation both *in vitro* and *in vivo* via the Frizzled receptor 1(Fzd-1) [14]. Hence, Wnt4 was analyzed in this study. In addition, Hua *et al.* have found that Wnt4 signaling induced VSMC proliferation and was associated with intimal thickening by regulating miR-126 [16]. The *Wnt4* gene plays a pivotal role in vascular occlusive disease and our current data suggest that miR-24 may ease this effect by reducing Wnt4 expression.

β-Catenin is a key molecule of the Wnt4 pathway, which could enter into the nucleus and activate the downstream signaling molecules [23]. Generally, the amount of free β-catenin in un-stimulated VSMCs is low with two available mechanisms. On one hand, the complex APC-Axin-GSK-3β, consisting of adenomatous polyposis coli (APC), Axin, and glycogen synthase kinase-3β (GSK-3β), could degrade the extra free β-catenin [15]. On the other hand, the free β-catenin in the cytoplasm could also bind the cytoplasmic tail of transmembrane proteins-cadherins [15,23], which could mediate cell-cell contacts and form adherens junctions. However, β-catenin would escape proteasomal degradation from the complex APC-Axin-GSK-3β and be released after the activation of Wnt4 signaling pathway [15,16,18,23]. Then, the free β-catenin would translocate to the nucleus and bind to members of the transcription factor family, including the T-cell factor (TCF)/lymphoid enhancer factor (LEF), regulating the expressions of downstream signaling molecules, including p21, Cyclin D1, and MMP [15]. Quasnichka *et al.* have indicated that β-catenin/T-cell factor signaling could regulate VSMC proliferation by modulation of the expression of Cyclin D1 and p21 [15]. Furthermore, Wang *et al.* also suggested that β-catenin andTCF-4 played a key role in vascular remodeling by inhibiting VSMC apoptosis and promoting proliferation [23]. The data from the current study support these previous findings. Briefly, β-catenin sharply increased in diabetic rats’ carotid arteries after a balloon injury, but it was reduced after up-regulation of miR-24. This tendency was consistent with Wnt4. The underlying mechanism of the anti-proliferative effects of miR-24 was associated with the Wnt4/β-catenin signaling pathway.

The exact mechanism through which Wnt4 signaling influences the complexes of APC-Axin-GSK-3β and cadherins to free β-catenin after binding to its receptor, Fzd-1, is unknown. Hua *et al.* have found that Dvl-1 may mediate a Wnt4-related VSMC proliferation signaling pathway [16,18]. Dvl-1 is an essential protein in the Wnt4 signaling pathway. Elevated Wnt4 interacts with Fzd-1 by binding to an N-terminal cysteine-rich-domain. The signal is then transduced into the cell through an internal sequence of Fz, which binds directly to the PDZ (postsynaptic density-95/discs large/zonula occludens-1) domain of the cytoplasmic protein Dvl-1 [24]. Dvl-1 then transduces the signal to downstream components [25]. We hypothesize that the activated Dvl-1 may influence the functional state of the complex APC-Axin-GSK-3β and cadherins through an uncovered mechanism, resulting in a rapid increase of free β-catenin in the cytoplasm. The change in Dvl-1 in our experiment supports this hypothesis, which strongly suggests that Dvl-1 participated in the Wnt4/β-catenin signaling pathway.

**Figure 7:**
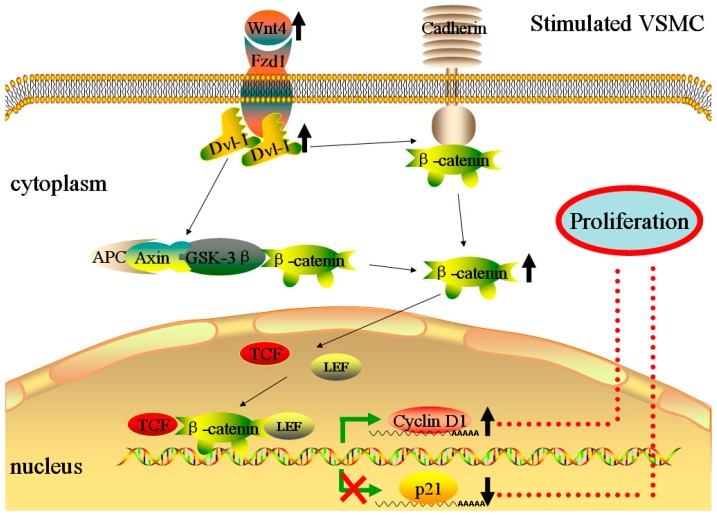
The mechanism of stimulated VSMC proliferation through the Wnt4/Dvl-1/β-catenin signaling pathway. External stimuli could increase the expression of Wnt4 and the elevated Wnt4 activate the cytosolic protein Dvl-1 by interacting with its receptor, Frizzled receptor 1 (Fzd-1). Then, the activated Dvl-1 may influence the functional state of the complex APC-Axin-GSK-3β (consisting of adenomatous polyposis coli (APC), Axin, and glycogen synthase kinase-3β) and cadherins, resulting in the rapid increase of free β-catenin in the cytoplasm. The free β-catenin would translocate to the nucleus and bind members of the T-cell factor (TCF)/lymphoid enhancer factor (LEF) transcription factor family, regulating the expressions of downstream signaling molecules, including p21 and Cyclin D1.

Cyclin D1 and p21, two key cell cycle-associated genes, would be regulated by the transcription complex β-catenin /TCF/LEF after free β-catenin translocates to the nucleus [26,27]. Cyclin D1 is a major promoter [26]. P21 is a cyclin-dependent kinase inhibitor (CKIs) and has a promoting effect on VSMC proliferation at low levels, but at higher levels displays an anti-proliferative effect [28]. Previous studies in other areas have shown that the expression of Cyclin D1 and p21 were regulated by a β-catenin/TCF-related signaling pathway [26,27]. Furthermore, Quasnichka found that the expressions of these two key cell cycle-associated genes in VSMC proliferation were also regulated by this signaling pathway [15]. In our experiment, both gene and protein levels of Cyclin D1 were in agreement with the change in β-catenin in diabetic rat carotid arteries after a balloon injury. However, p21 was completely opposite. Figure 7 shows a summary of the mechanism of the Wnt4/Dvl-1/β-catenin signaling pathway in stimulated VSMCs. At the same time, some limitations at this point also should be recognized. Because the direct regulation of p21 and Cyclin D1 by Wnt4 signaling has not been explored in this paper, other mechanisms could also be involved in the process of neointimal hyperplasia. In a word, further experiments, such as using the inhibitors or mimics of Wnt4 signaling related molecules, should be performed for a deep understanding of the precise mechanism.

The remarkable feature of vascular RS is neointimal hyperplasia, which is mainly caused by VSMC proliferation and EC dysfunction [29]. The existing treatments (such as DES) for vascular occlusive disease lack discrimination for VSMCs and ECs, which delays re-endothelialization, increasing the risk of late thrombosis following angioplasty [30]. Potential therapeutic approaches for vascular RS may arise by using special strategies that could inhibit VSMC proliferation while preserving endothelial function [29,30]. miR-24 is VSMC-enriched and our unpublished data also indicated that the effect of miR-24 is mainly due to VSMCs, but not ECs, which may be a huge advantage of using miR-24 to reduce RS. Lastly, genetic therapy has become a promising therapeutic approach to treat and heal cardiovascular diseases [31,32]. The delivery of miR-24 into diabetic rat carotid arteries via recombinant adenovirus vector is done to alleviate VSMC proliferation and neointimal hyperplasia and is just one type of gene therapy used in our present study.

In summary, we have shown that miR-24 adenoviral transfection could attenuate VSMC proliferation and neointimal hyperplasia in the diabetic rats after vascular injury by regulating the expressions of Cyclin D1 and p21 through inhibition of the Wnt4 signaling pathway. Thus, miR-24 may constitute a new therapeutic target for vascular RS.

## 4. Materials and Methods

### 4.1. Animals

Male Sprague-Dawley (SD) rats, eight weeks old (approximately 170 g), were purchased from China Three Gorges University (CTGU), Yichang, China. Animals received a standard diet and free access to water. The procedures for experiments and animal care were approved by the Animal Care and Use Committee of CTGU and conformed to the Guide for the Care and Use of Laboratory Animals by the National Institutes of Health (NIH Publication No. 80-23).

### 4.2. Streptozotocin (STZ)-Induced Diabetic Rats

Sixty male SD rats weighing 170 g were used for the current study. Rats were randomized into either a high-fat diet group (diabetic rat group, *n* = 50) or a control group (normal rat group, *n* = 10) and housed at 23 ± 2 °C under a cycle of 12 h light/12 h darkness with free access to food and water. The rats in the high-fat diet group were fed a high-fat diet (20% lard stearin, 10% sucrose, and 0.1% bile salt were added to the normal diet) for four weeks and then intraperitoneal injection streptozotocin (STZ, 30 mg/kg, dissolved in citrate buffer, pH 4.5; Sigma, St. Louis, MO, USA) for another week [33]. Rats in the normal group were fed standard rat chow and received an intraperitoneal injection of the same volume of citrate buffer. Animals had free access to diet and water throughout the experiment. Diabetes was considered to be induced once the blood glucose levels after 12 h of fasting ≥16.7 mmol/L [34].

### 4.3. Construction of miR-24 Expression Adenoviral Vector

The process of constructing the adenoviral vector has been described in our previously published work [35]. In brief, the precursor DNA of Rno-miR-24 (MI0000298) was synthesized by Genechem (Shanghai, China). The AdMax system (Microbix Biosystems, Toronto, ON, Canada) was used to generate the adenovirus expressing miR-24 (Ad-miR-24-GFP) or expressing Scramble (Ad-Scramble-GFP). HEK293 cells were used to package the generated adenoviruses. Viral titer was routinely concentrated to nearly 1 × 10^9^ PFU/mL determined by plaque assay.

### 4.4. Diabetic Rat Carotid Artery Balloon Injury Model and Adenovirus Transduction

One week before surgery, the 48 STZ-induced diabetic rats (350–400 g) were assigned into four groups randomly and equally: sham group (*n* = 12); carotid artery balloon injury with phosphate buffered saline treatment group (PBS group; *n* = 12); carotid artery balloon injury transfected with adenovirus expressing Scramble-GFP group (Ad-Scramble group; *n* = 12); and carotid artery balloon injury transfected with adenovirus expressing miR-24-GFP group (Ad-miR-24 group; *n* = 12). The procedure for rat carotid artery balloon injury model has been described previously [5]. In brief, animals were anesthetized by sodium pentobarbital (30 mg/kg, Sigma) via intraperitoneal injection and then intravenous injected with 100 U/kg of heparin sodium. The internal, external and common carotid arteries on the left side were also exposed sequentially. The distal external carotid artery was then partially cut with microscissors. Meanwhile, microvascular clips were also used to interrupt the blood flow temporarily by ligation of the internal and common carotid arteries. A balloon catheter (balloon diameter 1.25 mm, Medtronic, Minneapolis, MN, USA) was inserted into the common carotid through the external carotid cut. The balloon was inflated and passed three times with rotation. For adenovirus transduction, a 100 μL solution of Ad-Scramble (1 × 10^9^ PFU/mL), Ad-miR-24 (1 × 10^9^ PFU/mL) or PBS was infused into the injured common carotid artery segment and incubated for approximately 30 min. After adenovirus transduction, the next step is to ligate the external carotid artery and restore blood flow by loosening microvascular clips on the internal and common carotid arteries. Two weeks later, the rats were euthanized and the tissues were harvested for detection. The sham group did not undergo the balloon insertion procedure.

### 4.5. Assessment of Adenovirus Transfection Efficiency

To demonstrate the efficiency of adenovirus delivery into the carotid artery, Ad-Scramble and Ad-miR-24 were both labeled with GFP. Successful adenovirus transfection was evidenced by green fluorescence under fluorescence microscopy (Olympus, Nanjing, China).

### 4.6. Histologic Examination

Two weeks after operation, the control and injured carotid arteries were fixed in 4% paraformaldehyde for one week and then embedded in paraffin. Five round cross-sections (approximately 4 μm thickness) were cut from the paraffin and stained with hematoxylin-eosin (HE). For morphologic analysis of neointimal formation, Image-Pro Plus 5.0 professional image analysis software was used. The medial and intimal cross-sectional areas were measured and the intima/media ratios were calculated.

### 4.7. Assessment of VSMC Proliferation

To quantify the proliferative activity of VSMCs at the balloon injury sites, immunohistochemical staining of PCNA was performed [5]. Briefly, the carotid sections were incubated with anti-PCNA (dilution 1:100; Biotechnology Inc., Santa Cruz, CA, USA) antibody overnight at 4 °C, followed by incubation with a horseradish peroxidase-conjugated secondary antibody (Santa Cruz) for 1 h at room temperature. PBS substituted for the primary antibodies were used as negative controls. Lastly, color development was achieved with diaminobenzidine, and hematoxylin was applied as a counterstain prior to cover-slipping. Data are represented as a PCNA labeling index (LI), defined as the percentage of total cells within a given area positive for PCNA.

### 4.8. Quantitative Real-Time PCR (qRT-PCR) Analysis

The total RNA from the harvested carotid artery was extracted using TRIzol Reagent (Invitrogen, Carlsbad, CA, USA). Reverse transcription and qRT-PCR were performed [5]. The ABI Prism 7500 system (PE Applied Biosystems, Foster City, CA, USA) was used to amplify and detect the specific products. The mirVana™ qRT-PCR miRNA detection kit (Ambion Inc., Austin, TX, USA) was used to measure miR-24 levels and U6 as an internal control, 18S ribosomal RNA as internal control for Cyclin D1, p21 and GAPDH as internal control for PCNA, Wnt4, Dvl-1 and β-catenin. The method of 2^−ΔΔ*C*t^ was used. The following sequence-specific primers were used to amplify gene products: miR-24, F: 5’-TGCGCTGGCTCAGTTCAGCAGG-3’, R: 5’-CCAGTGCAGGGTCCGAGGTATT-3’; U6, F: 5’-CGCTTCGGCAGCACATATAC-3’, R: 5’-AAATATGGAACGCTTCACGA-3’; p21, F: 5’-AGGATCCATGTCAGAACCGGCTGG-3’, R: 5’-CAGGATCCTGTGGGCGGATTAGGG-3’; CyclinD1, F: 5’-AGTAGCAGCGAGCAGCAGAGT-3’, R: 5’-TTCATCTTAGAGGCCACGAA-3’; 18S ribosomal RNA, F: 5’-CGCGGTTCTATTTTGTTGGT-3’, R: 5’-CTTCAAACCTCCGACTTTCG-3’; PCNA, F: 5′-GACACATACCGCTGCGATCG-3′, R: 5′-TCACCACAGCATCTCCAATAT-3′; β-catenin, F: 5′-TGCAGCGACTAAGCAGGA-3′, R: 5′-TCACCAGCACGAAGGACA-3′; Dvl-1, F: 5’-CCTTCCATCCAAATGTTGC-3′, R: 5’-GTGACTGACCATAGACTCT-3′; Wnt4, F: 5’-TCAGGTTGGCCACGCACTAAAGGAG-3′, R: 5’-AGTCTGGACTTGGCTCCAGGTACAC-3′; GAPDH, F: 5′-ACCACAGTCCATGCCATCAC-3′, R: 5′-TCCACCACCCTGTTGCTGTA-3′.

### 4.9. Western Blotting Analysis

To determine the protein levels of PCNA, Wnt4, Dvl-1, β-catenin, Cyclin D1, p21 and GAPDH in injured carotid arteries, protein extraction and Western blotting analyses were performed [5]. Briefly, rat common carotid arteries were harvested for total protein extraction. The BCA protein assay (Pierce, Rockford, IL, USA) was used to measure protein concentration. A total of 20 μg of protein was separated on NuPAGE Novex 4-12% Bis-Tris Gel (Invitrogen) and transferred to a PVDF membrane. Then 5% non-fat dry milk were used to incubate the membranes for approximately 2 h at room temperature. Next, the above blots were incubated with primary antibodies overnight at 4 °C. PCNA (dilution 1:500; Santa Cruz), Wnt4 (dilution 1:500; Cell Signaling, Beverly, MA, USA), Dvl-1(dilution 1:1000; Santa Cruz), β-catenin (dilution 1:500; Cell Signaling), Cyclin D1 (dilution 1:500; Santa Cruz, CA, USA), and p21 (dilution 1:500; BD Biosciences Pharmingen). After washing three times, incubate these above blots with secondary antibodies (Pierce) for another 2 h at room temperature. Enhanced chemiluminescence (ECL) detection kits (Thermo, Rockford, IL, USA) were used to visualize these protein bands. The expression level of GAPDH served as a loading control and was approached to normalize the densities of different samples.

### 4.10. Statistical Analysis

All statistical analysis was performed with SPSS 19.0 software (SPSS Inc., Chicago, IL, USA). The data were presented as the mean ± SD. Statistical comparison was carried out with three or more groups using one-way analysis of variance (ANOVA) followed by Student-Newman-Keuls *post hoc* tests. The value of *p* < 0.05 was considered to be statistically significant.
